# Pilot Study: Detection of Gastric Cancer From Exhaled Air Analyzed With an Electronic Nose in Chinese Patients

**DOI:** 10.1177/1553350618781267

**Published:** 2018-06-18

**Authors:** Valérie N. E. Schuermans, Ziyu Li, Audrey C. H. M. Jongen, Zhouqiao Wu, Jinyao Shi, Jiafu Ji, Nicole D. Bouvy

**Affiliations:** 1 Maastricht University Medical Centre, Maastricht, Netherlands; 2 Beijing University Cancer Hospital & Institute, Beijing, China; 3 NUTRIM School of Nutrition and Translational Research in Metabolism, Maastricht, Netherlands

**Keywords:** gastric surgery, surgical oncology, evidence-based medicine/surgery

## Abstract

The aim of this pilot study is to investigate the ability of an electronic nose (e-nose) to distinguish malignant gastric histology from healthy controls in exhaled breath. In a period of 3 weeks, all preoperative gastric carcinoma (GC) patients (n = 16) in the Beijing Oncology Hospital were asked to participate in the study. The control group (n = 28) consisted of family members screened by endoscopy and healthy volunteers. The e-nose consists of 3 sensors with which volatile organic compounds in the exhaled air react. Real-time analysis takes place within the e-nose, and binary data are exported and interpreted by an artificial neuronal network. This is a self-learning computational system. The inclusion rate of the study was 100%. Baseline characteristics differed significantly only for age: the average age of the patient group was 57 years and that of the healthy control group 37 years (*P* value = .000). Weight loss was the only significant different symptom (*P* value = .040). A total of 16 patients and 28 controls were included; 13 proved to be true positive and 20 proved to be true negative. The receiver operating characteristic curve showed a sensitivity of 81% and a specificity of 71%, with an accuracy of 75%. These results give a positive predictive value of 62% and a negative predictive value of 87%. This pilot study shows that the e-nose has the capability of diagnosing GC based on exhaled air, with promising predictive values for a screening purpose.

## Introduction

Gastric carcinoma (GC) is the fourth most common cancer worldwide.^1,2^ It is most prevalent in Eastern Asia, Eastern Europe, and South America. Up to 42% of the cases are in Eastern Asia (mainly in China), where the majority of the annual GC-related deaths occur.^2,3^ The high mortality rates in China are linked to late detection, which is partly explained by the lack of signs and symptoms in GC patients.^4^ In addition to that, the lack of efficient screening tools remains an unsolved issue in China, where the majority of the gastric cancer cases are already locally advanced or worse at time of diagnosis. This can also be seen in many Western countries where no national screening programs for gastric cancer exist, given the relatively low incidence of the disease. Locally advanced GC results in significantly decreased survival when compared with early-stage GC, which may yield 5-year survival rates of 90% after surgical resection, endoscopic submucosal dissection (ESD) or endoscopic mucosal resection (EMR).^6,7^ Therefore, early detection and treatment seems to be the only way to reduce mortality, complications, and costs associated with the disease.^8^

Nowadays, endoscopy with biopsy remains the most accurate way of diagnosing GC. Therefore, the Korean screening program is indicated for individuals older than 40 years, in whom an upper-gastrointestinal (GI) series or an endoscopy is performed every two years. However, because this is a costly and invasive method, it is not routinely used as a screening tool in China or most other countries in the world. Moreover, some subtypes of gastric cancer can be easily missed on gastroscopy.^9^ Other radiological screening tools such as the conventional double-contrast barium radiograph with photofluorography, the Japanese screening strategy, requires highly trained personnel and, thus, has not yet been implemented in other countries. Although blood biomarkers such as CEA, CA125, and CA199 have a certain association with the progression of the disease, their sensitivity and specificity are too low for screening purposes.^10,11^

Breath analysis has recently emerged as a promising tool to diagnose several types of cancer and was shown to have especially high sensitivity and specificity for cancers of the head and neck and for colon carcinoma.^12^ The human breath contains a complex mixture of almost 3000 different volatile organic compounds (VOCs) that enter the exhaled air via the alveolar-capillary membrane of the respiratory tract.^13^ These can be identified by means of gas chromatography and mass spectrometry, which is a highly sensitive but costly and time-consuming technique because it requires both specialized equipment and trained personnel.^14,15^ Another analysis method that is commonly used in the consumer goods analyses, in the monitoring of air quality and detection of chemical agents, is pattern recognition by electronic nose (e-nose) technologies.^16^ e-Noses use an array of chemical sensors, a signal transductor and finally multivariate data analysis for pattern recognition to classify the samples.^17^ e-Nose technology also has many biomedical applications that can be used in the diagnosis of colon and lung cancer and chronic obstructive pulmonary disease (COPD), and cancers of the head and neck.^12,18-20^ Given the promising results of this technology in the detection of other diseases, the aim of this pilot study was to investigate whether it was possible to distinguish gastric cancer patients from healthy controls based on their exhaled breath patterns.

## Materials and Methods

### Patient Population

The study was conducted in the Beijing Oncology Hospital in a period of three weeks. The experimental group included patients diagnosed with GC who were admitted to the hospital prior to undergoing surgical resection of the tumor. The study was approved by the medical ethical committee of the Beijing Oncology Hospital and was conducted in accordance with the Declaration of Helsinki. The exclusion criteria were; age younger than 18 years, cognitive impairment and the presence of a tracheostomy. Other exclusion criteria included the presence of any concurrent infection that was judged to possibly interfere with interpretation of the data and the presence of comorbidities such as COPD or other breathing difficulties, which made the breathing test difficult or impossible to execute.

The control group included family members of the patients in the experimental group who were screened by means of endoscopy and were found not to suffer from GC. Patients and controls eligible for this study were given oral and written information about the study at the department. Informed consent was obtained from all patients.

An extensive database containing basic demographic factors (age, sex, length, weight) of all the patients as well as the location of the measurement was prospectively created. A questionnaire was filled out by all patients to detect any of the following complaints; nausea, vomiting, acid reflux, heartburn, abdominal distension, loss of appetite, dysphagia, ructus, fatigue, hematemesis, blood in stools, abdominal discomfort, weight loss, or jaundice. Previous diagnostics of all participants were documented (gastroscopy, GI radio diagnostics, computed tomography [CT], magnetic resonance imaging, or other). For all patients included in the study, pathology results after surgical resection were documented: tumor location, tumor macroscopy, pathological differentiation (high, medium, or low grade), mucinous/signet ring appearance, TNM-staging based on CT and on ultrasound, CA125 level, and neoadjuvant therapy.

### Materials

This study used the Aeonose, which is a CE-certified e-nose device manufactured by The eNose Company located in Zutphen, the Netherlands. It is a proprietary software package Aethena. Details have been described by Kort et al.^21^ One eNose was purchased for the purpose of this study and was used to test all patients.

It contains 3 micro-hotplate metal-oxide sensors and a pump. The hotplates are alternatively heated and cooled during the measurement during which the sensors are exposed to the exhaled air. Reactions of the VOCs on the sensors’ surfaces alter their conductivity, which generates unique patterns.^22^ The airflow is controlled by a solenoid switch selecting between 2 different inlets, thereby facilitating an active airflow across all sensors. One inlet is connected to an active carbon filter to provide a baseline free from environmental influence; the other inlet is attached to the breathing tube. The disposable mouthpiece consists of a nonrebreathing T-valve with an active carbon filter attached to the inlet. During sampling, a nose clamp was placed on the nose of the participant to avoid entry of nonfiltered air. The first 2 minutes are used to flush out environmental influences from the lungs after which the exhaled air is measured for 3 minutes followed by a 4-minute recovery period. The built-in absorber is cleared for one minute (20s heating followed by 40s cooldown) to release the attached volatiles, after which the final measurement is performed under influence of the clean reference air. This process takes another 5 minutes. Thus, each single measurement comprises an adsorption and desorption step, each associated with specific chemical dynamics at the sensor’s surface.

### Study Design

Measurements were performed in the treatment room of the Department of Gastrointestinal Surgery in the Beijing Oncology Hospital. All participants were asked to inhale and exhale through the mouthpiece attached to the Aeonose for 5 minutes. The VOCs are analyzed within the e-nose and exported as numerical data. The main objective is not to define a specific VOC in the measurement, but rather to determine the pattern of resistance changes in the sensors caused by the absorption of the various VOCs in the breath of patients. This results in a graphic pattern specific for each disease.

### Statistical Analysis

Differences in baseline characteristics between the experimental and control groups were determined with the independent-sample *t* test, χ^2^ test, and Fisher exact test. The Aeonose measures the air composition every 20 seconds using two 32-step sinusoidal modulations of the sensor surface temperature, thus resulting in a vector of 64 values every 20 seconds for each of the three sensors. These numerical codes were exported and normalized to minimize and eliminate interdevice differences. The normalized data of qualified samples are then used to train an artificial neural network (ANN). The ANN is a computational system comparable to the neural network of the human brain and thereby enables itself to learn.

**Figure 1.**
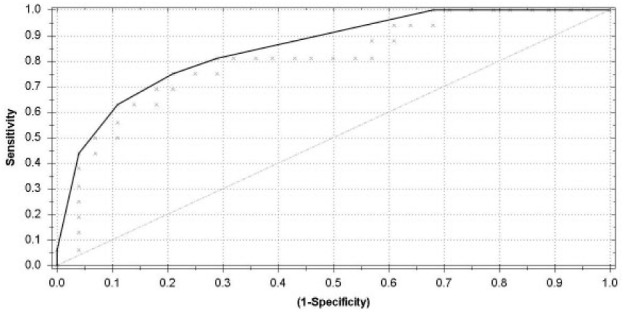
The receiver operating characteristic curve illustrates the different sensitivities and specificities with altered thresholds of both the best fit of the data (dark gray line) and the data for double cross-validation (light gray line). The area under the curve is 0.83.

Training groups are selected carefully to prevent separation shown from effects other than the disease (eg, separation on gender, age, use of medication). The network is being trained specicifically on the disease. Other effects should level out. Cross-validation is a technique that helps in this respect. These results were presented in a scatter plot and receiver operating characteristic (ROC) curve. A leave-one-out analysis was performed for final validation when plotting the negatives and positives against the predictive value (Figure 1) to detect outliers. Data were analyzed with Aethena 2.0 version 0.92.

## Results

Within a period of three weeks, 44 individuals were included (patients, n = 16; controls, n = 28). All gastric cancer patients underwent a resection after which the gastric cancer was proven by histopathology. The baseline characteristics of the two groups are listed in Table 1. Baseline characteristics in the group only differed significantly in age, where the average age of the control group was 37 years and that of the diseased group 57 years. This is most likely explained by the fact that younger, healthy family members accompany the patient to the hospital. The sex between the groups did not differ significantly; weight and length were similar. The fact that there was no significant difference in the number of symptoms between patients and controls is striking and further underlines the difficulty of diagnosing gastric malignancy because of the lack of symptoms.

**Table 1.** Baseline Characteristics of Controls and Gastric Cancer Patients.

	Controls (n = 28)	Disease (n = 15)	*P* Value
Sex (M/F)	11/17	11/4	.330
Age	37	57	.000
Weight (kg)	65	68	.374
Length (cm)	166	166	.926

All participants filled out questionnaires concerning symptoms possibly related to GI disease. Weight loss was the only symptom reported that differed significantly between the 2 groups, where GC patients had more complaints of weight loss than controls (Table 2).

**Table 2.** Self-reported Presence of Gastrointestinal Symptoms in Control and Gastric Cancer Patients.

	Control (n = 28)	Disease (n = 15)	*P* Value
Abdominal distension	15	4	.072
Abdominal pain	15	9	1.000
Ructus	8	9	.129
Heartburn	11	5	.752
GERD	12	4	.342
Dysphagia	3	2	.530
Loss of appetite	6	1	.391
Weight loss	2	5	.040
Fatigue	10	5	1.000
Nausea	6	1	.243
Vomiting	4	2	1.000
Hematemesis	0	0	—
Melena	4	1	.643
Jaundice	1	0	1.000
Total number of symptoms/patient	3.1	3.6	.531

Abbreviation: GERD, gastroesophageal reflux disease.

**Figure 2.**
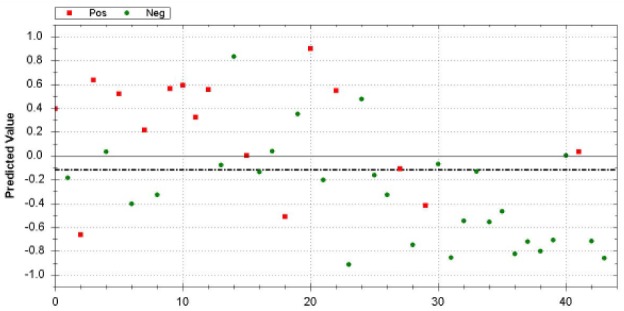
Scatter plot of gastric cancer patients (positive [Pos]: red dots) and healthy controls (negative [Neg]: green dots). Values greater than −0.31 are scored as positive for gastric cancer.

Of the 44 included patients, 13 were proved true positives and 20 true negatives. The ROC curve showed a sensitivity of 81% and a specificity of 71% in differentiating between GC and healthy controls, with an overall accuracy of 75%. The Matthews Correlation Coefficient was calculated to show the binary classification between the patients and controls, which was 0.51. A positive predictive value of 62% and a negative predictive value of 87% was found. A leave-one-out analysis was performed for final cross-validation. Figure 2 is a scatter plot of individual predictive values with a best fit of the data analyzed by the ANN. The threshold was set to −0.12. Cross-validation data are presented in Figure 1.

When looking at the scatterplot, several outliers can be seen. Three patients (red dots) are seen amid the controls. One of these was a patient with a gastrointestinal stromal tumor (GIST) tumor identified on pathology. The location of the tumor did not seem to affect the outcome of the test.

Three control patients were classified by the e-nose as if they had gastric cancer; these controls will be asked to undergo an endoscopy to determine the presence of GC.

## Discussion

It has been shown that several diseases can be detected in exhaled breath. Until recently, breath analysis studies have mainly focused on the technique of gas chromatography and mass spectrometry. This technique has several disadvantages because sample preparation and data analysis are time-consuming and require highly trained personnel; it is, therefore, very costly. The e-nose has emerged as an alternative method to analyze the composition of breath samples and does so with pattern recognition software that allows the researcher to link specific patterns with a wide range of pathologies. This pilot study examined the ability of the e-nose to recognize the presence of GC in preoperative patients, in their exhaled air, and to distinguish them from healthy controls. Our results suggest that the e-nose is a very promising screening tool; further studies in a larger patient population are therefore recommended. Several limitations should be considered when interpreting our study results, of which the case-controlled nature and small sample size are the 2 most important. Furthermore, only Chinese patients were included in this pilot study. Even though this population is an accurate representative of the Eastern Asian population, we acknowledge that the applicability of our results to the Western populations is unknown. The control group consisted of family members of the patients included in this study, which allows for correction of the influence of the background of the patient/control on the breath composition. However, in further investigations, a general population should be used as a control group to further elucidate its efficacy as a screening tool.

Previous literature has proven that the e-noses are able to distinguish between different types of oncological and nonmalignant diseases.^23-25^ Shehada et al^9,25^ have performed the only two other studies concerning detection of gastric cancer in exhaled breath, to our current knowledge. These studies distinguished early and late stages of GC from dyspeptic controls proven not to have GC.^25^ This resulted in a sensitivity of 87% in 30 patients and 77 controls^9^ and a sensitivity of 87% in 149 gastric cancer patients versus 129 controls.^25^ These good results in sensitivity in large populations are promising results that show the possibility of detecting gastric cancer in exhaled breath. Silicon nanowire sensors were used; these require a coating that were chosen based on results from earlier mass spectrometry studies for gastric cancer, although in this study, patients breathed in a collecting bag to collect breath samples. These are stored in tubes that are then refrigerated and transported for analysis, which requires less time and material and fewer personnel. Moreover, in the breath test performed by the silicon nanowire sensors, patients were required to fast and withhold alcohol and cigarettes two hours before the breath tests. The advantage of the e-nose used in this study is the real-time analysis. By immediately analyzing the exhaled air in the e-nose during and after the breath test, no storage and additional testing is used.

The most important advantage of the e-nose is evident: the test is completely noninvasive. The 100% enrollment rate of this pilot study implies that breath testing can be widely accepted as a future screening method. With three false positives and 13 true positives, three false negatives and 20 true negatives in our group of 44 individuals, the e-nose proved to have a sensitivity of 81% and a specificity of 71%. Postoperative pathological examination proved one of the false negatives to be a GIST tumor, which may explain the deviating results. The location of the tumor did not seem to affect the outcome of the test, even though the sample size might have been too small to detect the association between tumor site and test outcome. The other 2 false-negative breath samples were obtained from measurements that were interrupted shortly because the patients were short of breath, which may have altered the outcome. The high sensitivity of the e-nose can be explained by the broad spectrum of VOC reactions on multiple sensors at different temperatures and by the use of an ANN, which recognizes patterns more sensitively with every inclusion through a self-learning system. A study by Dragonieri et al^26^ suggested that the overall VOC profile does not differ by age group or gender.

Moreover, the fact that the GIST tumor did not cluster with those of the other GC patients, of which pathological investigation proved to be adenocarcinoma might show the ability of the e-nose to differentiate between types of GCs. Our and previous findings suggest that use of the e-nose enables the screening of multiple diseases with a single measurement.^27^ However, exhaled breath patterns may be influenced by environment and lifestyle (diet in particular), although this was not taken into account here, and the results showed a good sensitivity. Further investigation and validation studies in different patient populations are necessary.

In current medical practice, endoscopy may be suggested in patients with gastric complaints or a positive family history, resulting in many negative diagnostic tests. We believe that the e-nose may find its role in medical practice as a screening technique and may well be used in combination with, or instead of, endoscopy as a first-line diagnostic tool for GC. By selecting patients based on the outcome of the e-nose, a high-risk patient population can be targeted for further examination with endoscopy and biopsy. Therefore, by reducing the need of invasive examinations, the e-nose may substantially reduce health care costs.

When compared with endoscopy and biopsy, the accuracy of the Aeonose to differentiate between GC patients and healthy controls in this pilot study remains relatively low. On the other hand, this pilot study of GC detection in exhaled breath already shows more potential than the reported accuracy of all blood tests investigated for GC.^28^ Therefore, and also because of the self-learning capacity of the ANN involved, which will increase the sensitivity and specificity tremendously, the Aeonose is believed to be a promising tool for future screening purposes. Based on the current results, a prospective cohort study with a larger and more varied population is planned to further train the ANN in recognition of gastric cancer. After this, a blinded validation should take place in a group to further explore and determine the diagnostic accuracy of the e-nose.

## Conclusions

This pilot study shows that the e-nose has the capability to distinguish gastric cancer patients from healthy controls, based on samples from exhaled air.
